# Vitamin D levels in an Australian population

**DOI:** 10.1186/1471-2458-14-1001

**Published:** 2014-09-26

**Authors:** Tiffany K Gill, Catherine L Hill, E Michael Shanahan, Anne W Taylor, Sarah L Appleton, Janet F Grant, Zumin Shi, Eleonora Dal Grande, Kay Price, Robert J Adams

**Affiliations:** School of Medicine, Faculty of Health Sciences, The University of Adelaide, Level 3, 122 Frome St, Adelaide, SA 5000 Australia; Rheumatology Department, The Queen Elizabeth Hospital, Woodville Rd, Woodville, SA 5011 Australia; The Health Observatory, The Queen Elizabeth Hospital, The University of Adelaide, Adelaide, SA 5005 Australia; Rheumatology Department, Southern Adelaide Health Service, Repatriation General Hospital, Daws Rd, Daw Park, SA 5042 Australia; Flinders University, Bedford Park, SA 5041 Australia; Population Research and Outcome Studies, Discipline of Medicine, Faculty of Health Sciences, The University of Adelaide, Adelaide, SA 5005 Australia; School of Nursing and Midwifery, University of South Australia, Adelaide, SA 5000 Australia

**Keywords:** Vitamin D, Population, Cohort study, Risk factors, Demographic factors

## Abstract

**Background:**

Levels of vitamin D in the population have come under increasing scrutiny, however there are only a few studies in Australia which measure levels in the general population. The aim of this study was to measure the levels of vitamin D within a large population cohort and examine the association with seasons and selected demographic and health risk factors.

**Methods:**

A longitudinal cohort study of 2413 participants in the northwest suburbs of Adelaide, South Australia conducted between 2008 and 2010 was used to examine serum levels of 25-hydroxy vitamin D (25(OH)D) in relation to demographic characteristics (age, sex, income, education and country of birth), seasons, the use of vitamin D supplements and selected health risk factors (physical activity, body mass index and smoking). Both unadjusted and adjusted mean levels of serum 25(OH)D were examined, as were the factors associated with the unadjusted and adjusted prevalence of serum 25(OH)D levels below 50 and 75 nmol/L.

**Results:**

Overall, the mean level of serum 25(OH)D was 69.2 nmol/L with 22.7% of the population having a serum 25(OH)D level below 50 nmol/L, the level which is generally recognised as vitamin D deficiency. There were significantly higher levels of 25(OH)D among males compared to females (t = 4.65, p < 0.001). Higher levels of 25(OH)D were also measured in summer and autumn compared with winter and spring. Generally, mean levels of 25(OH)D were lower in those classified as obese. Smokers and those undertaking no or less than 150 minutes/week of physical activity also had lower levels of serum vitamin D. Obesity (as classified by body mass index), season and undertaking an insufficient level of physical activity to obtain a health benefit were significantly associated with the prevalence of vitamin D deficiency.

**Conclusions:**

Vitamin D deficiency is prevalent in South Australia, affecting almost one quarter of the population and levels are related to activity, obesity and season even when adjusted for confounding factors. Improved methods of addressing vitamin D levels in population are required.

## Background

Vitamin D plays an important role in the formation of bone and low levels of vitamin D have been associated with the development of rickets and osteoporosis [1, 2]. There is also evidence to suggest that vitamin D deficiency is associated with mortality [3] and a wide range of other conditions including type 2 diabetes [4], gestational diabetes [5], cardiovascular disease [6], rheumatoid arthritis [7], chronic obstructive pulmonary disease [8], type 1 diabetes, some cancers (for example, colorectal, prostate, breast) and multiple sclerosis [1], muscle weakness [1] as well as chronic kidney disease [9], schizophrenia and depression [1]. However, these studies are often cross sectional in nature and undertaken under varying conditions, making comparisons difficult and limiting the strength of the evidence.

Generally, the primary source of vitamin D is from ultraviolet exposure via sunlight or from food or supplements [1]. Vitamin D is then converted to 25-hydroxyvitamin D in the liver. Serum level of 25-hydroxyvitamin D (25(OH)D) provides an indication of overall vitamin D status [1]. Levels of vitamin D vary according to season [1] and it is acknowledged that vitamin D deficiency is generally high within the population and varies according to the population under examination [1]. Various cutoffs have also been reported with deficiency defined as <25 nmol/L 25(OH)D, insufficiency as 25-50 nmol/L, and sufficiency >50 nmol/L by Lau et al. [5]. Deficient has also been defined as <27.5 nmol/L, insufficient as 27.5-49.9 nmol/L and suboptimal as 50- < 75 nmol/L by Green-Finstone et al. [10]. In South Australia (SA), the optimal range defined by SA Pathology is 60-160 nmol/L derived from levels required to suppress parathyroid hormone and bone turnover markers [11]. However, vitamin D deficiency has more recently been considered to be less than 50 nmol/L [1, 12].

Vitamin D levels have been measured in association with specific disease groups and mortality [1–9], however there are fewer studies which examine the prevalence of vitamin D deficiency at a population level using large samples. Internationally, research conducted in Canada, the United States, Great Britain, New Zealand and Denmark [10, 13–17] have examined vitamin D deficiency in population-based studies, with the prevalence of vitamin D levels < 50 nmol/L ranging between 18% and 52%, although different cutoff values for vitamin D deficiency are reported in some of these studies. International comparisons can also be difficult due to differences in factors such as testing methods, latitudes, season of testing, age range tested and population composition. Thus specific Australian studies also have relevance.

A study conducted in south-east Queensland at the end of winter among 126 adults aged 18–87 years living in the community identified that 10.2% of the participants had serum 25(OH)D levels below 25 nmol/l, which was considered to be deficient. A further 32.3% had a level of between 25 nmol/l and 50 nmol/l, which was defined as insufficient [18]. In Tasmania, among a community control group of 272 adults, 8.8% had 25(OH)D levels up to 25 nmol/L, with 14.5% having 25(OH)D levels 26-40 nmol/L and a further 25.3% between 41-50 nmol/L [19]. A cross-sectional, population-based study of women aged 20–92 years (n = 861) in Geelong, Victoria demonstrated that 11.3% and 7.2% of the population had serum 25(OH)D levels of <28 nmol/L in winter and overall respectively and 43.2% and 30.0% had levels <50 nmol/L, in winter and overall, respectively [20]. A comparison of the previous two studies [19, 20] and another in southeast Queensland [21] demonstrated that, among females less than 60 years of age, the prevalence of vitamin D insufficiency <50 nmol/L was 67.3% in Tasmania, 40.5% in Queensland and 37.4% in the Geelong area. While season and latitude both had an impact on vitamin D levels, less than one fifth of the variation was explained by these factors highlighting the importance of behavioural factors [22]. The largest study has been conducted by Daly et al. [23] and examined vitamin D levels obtained from 11,247 samples collected Australia wide. This study demonstrated that vitamin D deficiency (<50 nmol/L) was common in Australian adults aged 25 years and over (31%) and that those most at risk were women, the obese, the elderly, those not meeting physical activity guidelines and those with a non-European background [23]. However, the blood used for serum 25(OH)D testing was taken at only one time point, between May and July 2000 in SA, which is the end of autumn and the beginning of winter. Daly et al. [23] point out that the pooling of results obtained from all states of Australia could be misleading and that by testing blood that was collected in 1999/2000, there may have been changes in vitamin D status since that time.

Adelaide, the capital of South Australia lies at a latitude 36 degrees S. Thus, while it is not below the 42 degrees of latitude which has been shown to be the level where cutaneous production of vitamin D does not occur [24], vitamin D will certainly be synthesized at varying rates, throughout the year, depending on the season and will be in contrast to previous Australian studies undertaken in different locations. Work undertaken by Morris et al. [25] as early as 1984 demonstrated a significant seasonal variation in 25(OH)D levels in SA, tested as part of routine assays. However, to date there have been no large scale epidemiological studies to examine vitamin D levels specifically in this state. The advantages of this study are that it is solely focused on the population of Adelaide and involves a population cohort of over 2000 participants. Vitamin D levels were determined from samples taken across all seasons and as this is a longitudinal cohort study, the potential exists to examine levels of 25(OH)D in conjunction with a wide range of demographic, chronic disease, risk and social factors which have been collected over time. Information related to vitamin D supplementation is also available.

This paper reports the level of vitamin D deficiency and examines these values in conjunction with various demographic (age, sex, income, education) and health risk factors (body mass index, physical activity and smoking), season and the use of vitamin D supplements.

## Methods

This paper reports data collected as part of Stage 3 of the North West Adelaide Health Study (NWAHS). The NWAHS is a representative longitudinal study of 4056 randomly selected adults aged 18 years and over at the time of recruitment from the north-west region of Adelaide, the capital of SA. The sample region represents approximately half of the metropolitan area (population of approximately 1.3 million) and almost one-third of the population in SA (population of approximately 1.68 million), which has the second highest elderly population of all the Australian states and territories. The study commenced in 1999 to 2003 and Stage 2 was conducted between 2004 and 2006. Stage 3 was conducted between 2008 and 2010, with testing being undertaken across all months using identical methodologies.

Information was obtained from a computer assisted telephone interview (CATI), a self-completed questionnaire and a clinic assessment. Of the original cohort of participants (n = 4056), n = 2487 completed the clinic assessment in Stage 3, with n = 2710 completing the CATI survey and n = 2638 the self-complete questionnaire. The response rate for each assessment following completion of Stage 3 was 73.0% for the CATI survey, 71.1% for the written questionnaire and 67.0% for the clinic assessment.

Country of birth and smoking were determined from responses to the self-complete questionnaire. Gross annual household income prior to tax and highest level of education of participants were collected as part of the telephone questionnaire. The level of physical activity was determined from descriptions of physical activity type and time using the questions from the Active Australia survey [26]. This information was used to calculate whether respondents had achieved a sufficient level of physical activity to achieve a health benefit in the past week [26]. Sufficient physical activity was defined as a total of 150 minutes of walking, moderate or vigorous physical activity with vigorous activity weighted by a factor of two to account for its greater intensity [26]. Age was calculated from date of birth and the date of attendance at the clinic assessment and the season when blood was taken was determined from the month of the clinic assessment. Information relating to the use of vitamin D supplements within the past 12 months was asked as part of the Dietary Questionnaire for Epidemiological Studies (DQES, Cancer Council of Victoria), which was used in this study to assess diet and nutrition.

Height and weight were measured with standardized protocols. A wall mounted stadiometer measured height to the nearest 0.5 centimetres and weight was measured using calibrated scales to the nearest 0.1 kilograms. Body mass index (BMI) was then calculated (weight (kg)/height (m^2^)) [27].

Fasting blood samples were collected and analysed in real time by the NATA certified laboratories of SA Pathology in Adelaide. The vitamin D assay was initially measured (until April 2010) using the Enzyme Immunoassay method and was performed on a BEST 2000 automated ELISA system (Biokit). The interassay coefficient of variation (CV) was 5.6% for a mean of 33.3 nmol/L and 3.9% for a mean of 112.8 nmol/L. After April 2010, the assay was changed to an Automated Chemiluminescent assay and performed on an iSYS Automated Immunoassay system (IDS). The CV was 10.5% for a mean of 33.4 nmol/L, 7.0% for a mean of 80.3 nmol/L and 7.3% for a mean of 165.2 nmol/L. Overall, 90% of assays were conducted using the first test procedure. When the assays were changed the patient comparison gave a Passing-Bablock regression equation of y = -1.61 + 1.07x with a bias of -1.9 nmol/L indicating good agreement between the two assays. Not all participants in Stage 3 were willing or able to provide a blood sample, thus this analysis focuses on those respondents who provided blood for testing and for whom a vitamin D test was able to be performed (n = 2413).

In Stage 1, data were weighted by region (western and northern health regions), age group, sex and probability of selection in the household to the Australian Bureau of Statistics 1999 Estimated Resident Population and the 2001 Census data. Stage 3 was reweighted using the 2009 Estimated Resident Population, incorporating participation in the three components, whilst retaining the original weight from Stage 1 in the calculation. All analyses conducted in this paper are weighted to the population of the northern and western suburbs of Adelaide.

Ethics approval for the study was obtained from the Human Research Ethics committee of The Queen Elizabeth Hospital, Adelaide, South Australia and all participants provided informed consent.

Statistical analyses were conducted using SPSS Version 19 (IBM SPSS Statistics, New York, NY, USA) and Stata version 12 (StataCorp, College Station, TX, USA). Descriptive characteristics (means and proportions) are presented for all participants as are the proportions of participants with various levels of 25(OH)D. T-tests and chi square tests were used to determine significant differences in vitamin D levels between males and females. ANOVA determined significant differences in the mean age of those who did and did not take supplements. The unadjusted mean values of serum 25(OH)D were determined by age group, BMI, country of birth, season, income, education, smoking status, physical activity and taking vitamin D supplements. The mean levels of 25(OH)D were then adjusted for the above variables, all of which may act as confounders. The prevalence of males and females with vitamin D levels less than 50 nmol/L and less than 75 nmol/L was determined by age group, BMI, country of birth season, income, education, smoking status, physical activity and taking vitamin D supplements and multivariable logistic regression analysis was undertaken with all of the above variables in the model to determine independent predictors of vitamin D deficiency (serum 25(OH)D < 50 nmol/L) and vitamin D levels < 75 nmol/L.

## Results

Overall there were no significant differences in the proportion of males and females and within each age group for those who did, and did not, provide a blood sample for serum 25(OH)D testing. The mean age of those who provided a vitamin D sample was 50.6 years (SD 16.6, range 24–95). Overall, the mean level of serum 25(OH)D was 69.2 nmol/L (n = 2413, SD 26.4, range 14–286). Levels ≤ 220 nmol/L can be considered within the normal physiological range [28], thus within this cohort three participants may have toxic levels of serum 25(OH)D (data not shown). There was a significant difference between males (n = 1164, mean 72.5, SD 26.5) and females (n = 1249, mean 66.0, SD 25.9) in serum 25(OH)D (t = 4.65, p < 0.001).Of those who had serum 25(OH)D levels measured, only 4.1% reported that they took vitamin D supplements with the mean age of those respondents significantly higher (59.23 years) compared to those who did not respond or take supplements at all (50.18 years) or who took supplements but did not take vitamin D (50.38 years; F = 14.21, p < 0001). Overall 9.0% of those aged 75 years and over took vitamin D supplements compared to 0.9% of those aged 24 to 34 years, with the proportion increasing across all age groups.

The characteristics of those who provided a sample for vitamin D testing are presented in Table 1. Participants were primarily born in Australia, with approximately a third of both males and females classified as obese. A higher proportion of males were current smokers and a lower proportion of females undertook a sufficient level (≥150 minutes per week) of physical activity.

**Table 1:** **Descriptive characteristics of those who provided a vitamin D sample, by sex**

Variable	Male (n = 1164)	Female (n = 1249)
Age [mean ± SD]	49.6 ± 16.2	51.5 ± 16.9
BMI [mean ± SD]*	28.6 ± 4.8	28.5 ± 6.4
Underweight (%)	0.3	1.4
Normal (%)	21.9	30.8
Overweight (%)	45.9	32.5
Obese (%)	31.8	35.3
Country of birth (%)*		
Australia	72.9	73.8
Country other than Australia	27.1	25.9
Season (%)		
Summer (Dec-Feb)	18.5	23.6
Autumn (Mar-May)	23.9	21.5
Winter (Jun-Aug)	29.7	29.1
Spring (Sep-Nov)	28.0	25.8
Income (%)		
Up to $60,000	39.7	46.9
More than $60,000	51.8	41.7
Don’t know/not stated	8.5	11.4
Education (%)		
Some schooling	25.4	40.2
Completed high school	10.4	14.7
Apprenticeship/diploma	40.3	24.4
University or higher education	20.7	18.4
Refused/not stated	3.2	2.3
Smoking status (%)		
Current smoker	18.2	16.7
Ex/non smoker	80.4	82.0
Not stated	1.4	1.2
Physical activity (%)		
No activity	15.1	20.5
Insufficient activity	28.4	35.0
Sufficient activity	52.9	42.1
Not stated	3.6	2.4
Vitamin D supplement (%)		
Took vitamin D	2.0	6.1
Took supplements but not vitamin D	25.0	29.3
Did not take supplements/not stated	73.1	64.6

*Not stated category not reported n ≤ 10.

The weighting of data can lead to rounding discrepancies and totals not adding.

Due to the significant difference in mean serum levels between males and females, the analysis of each sex was undertaken separately. The unadjusted and adjusted mean serum levels of 25(OH)D for age group, BMI, country of birth, season, income, education level, smoking status, physical activity level and whether or not vitamin D supplements were taken are presented in Table 2. Both adjusted and unadjusted mean levels of serum 25(OH)D were higher in both summer (December, January, February) and autumn (March through May). Those with lower levels of education (a trade certificate or diploma) also had higher levels of 25(OH)D as did ex-smokers compared to current smokers. For both males and females, those who undertook no activity had lower mean vitamin D levels compared to those who had a sufficient level of activity (150 minutes or more of walking, moderate or vigorous activity during the week).

**Table 2:** **Mean (95% CI) level of vitamin D (nmol/L), by age group, BMI categories, country of birth, season, income, education, smoking status, activity level and vitamin D supplement status for males and females**

	Males				Females	
***Variables***	***n***	***Unadjusted***	***Adjusted*** ^***†***^	***n***	***Unadjusted***	***Adjusted***
**Overall population**	1164	72.5 (70.5-74.6)	69.1 (68.5-69.8)	1249	66.0 (64.2-67.8)	69.2 (68.5-69.9)
**Age (years)**						
24-34 years	252	70.0 (63.9-76.2)	68.1 (66.1-70.1)	248	65.1 (59.5-70.6)	67.1 (64.7-69.5)
35-44 years	254	72.8 (67.6-78.0)	69.1 (67.7-70.4)	256	64.9 (60.6-69.2)	68.6 (67.0-70.1)
45-54 years	241	70.2 (66.7-73.7)	66.4 (65.3-67.4)	245	63.0 (59.8-66.2)	66.8 (65.7-67.9)
55-64 years	188	73.8 (70.1-77.5)	70.2 (69.1-71.2)	202	67.2 (64.0-70.4)	70.6 (69.5-71.7)
65-74 years	122	78.5 (74.1-82.9)	72.5 (71.3-73.6)	141	67.6 (63.3-71.8)	72.7 (71.5-74.0)
75 years and over	108	74.0 (69.3-78.7)	72.1 (70.7-73.4)	157	71.1 (66.4-75.7)	72.4 (70.9-73.9)
BMI*						
Underweight	4	80.9 (62.2-99.7)	56.8 (52.2-61.5)	18	51.4 (32.9-69.8)	56.7 (53.1-60.2)
Normal	256	74.9 (70.9-78.9)	73.4 (72.1-74.7)	385	72.5 (68.8-76.1)	73.4 (72.2-74.7)
Overweight	534	74.7 (71.3-78.1)	70.8 (69.9-71.7)	406	66.3 (63.4-69.2)	71.4 (70.2-72.5)
Obese	370	67.8 (64.5-71.1)	64.0 (62.0-64.7)	440	60.7 (58.1-63.4)	64.0 (63.2-64.9)
Country of birth*						
Australia	848	73.7 (71.2-76.2)	69.8 (69.0-70.6)	922	66.4 (64.3-68.6)	70.0 (69.2-70.9)
Other country	315	69.5 (66.1-72.9)	67.3 (66.3-68.3)	323	64.9 (61.6-68.2)	67.0 (65.9-68.2)
**Season**						
Summer	215	84.1 (78.0-90.2)	77.1 (75.9-78.4)	295	71.3 (67.3-75.3)	76.4 (74.8-78.0)
Autumn	278	77.6 (73.7-81.4)	73.2 (72.2-74.2)	269	69.0 (65.5-72.4)	73.5 (72.5-74.5)
Winter	345	67.5 (63.5-71.4)	65.6 (64.7-66.5)	363	63.1 (59.8-66.3)	64.9 (63.9-65.8)
Spring	326	66.0 (63.4-68.6)	64.1 (63.1-65.0)	322	62.0 (58.4-65.7)	64.0 (63.1-64.9)
**Income**						
Up to $60,000	462	74.3 (71.3-77.4)	69.6 (68.7-70.5)	586	65.5 (63.0-68.0)	69.1 (68.3-70.0)
More than $60,000	603	71.3 (68.2-74.4)	68.9 (67.9-69.8)	520	65.6 (62.6-68.7)	68.6 (67.5-69.7)
Don’t know/not stated	99	71.8 (64.8-78.7)	68.2 (65.7-70.8)	143	69.7 (64.9-74.4)	72.1 (69.4-74.8)
**Education**						
Some schooling	296	74.1 (70.7-77.6)	69.2 (68.5-70.3)	502	67.0 (64.3-69.7)	69.9 (69.0-70.7)
Completed high school	122	73.7 (67.8-79.6)	68.9 (66.5-71.3)	184	66.8 (61.9-71.8)	70.0 (68.3-71.7)
Apprentice/diploma	469	75.1 (71.7-78.6)	70.9 (70.0-71.8)	305	63.8 (60.4-67.3)	70.3 (68.6-72.0)
University or higher education	240	66.8 (62.2-71.4)	66.6 (65.2-68.0)	230	65.9 (61.2-70.6)	66.2 (64.5-67.9)
Refused/not stated	38	60.7 (50.8-70.5)	62.7 (59.9-65.4)	28	67.6 (53.4-81.8)	64.9 (60.8-69.1)
**Smoking status**						
Non/ex	937	73.4 (71.1-75.7)	69.7 (69.0-70.3)	1025	66.5 (64.4-68.5)	69.9 (69.1-70.6)
Current	212	69.2 (64.4-73.9)	66.5 (64.5-68.5)	209	63.5 (59.7-67.3)	66.2 (64.8-67.7)
Not stated	16	66.9 (55.3-78.5)	70.7 (67.5-73.8)	15	70.8 (59.2-82.5)	66.9 (61.8-72.0)
**Physical activity**						
No activity	176	71.5 (65.3-77.6)	65.1 (63.6-66.7)	256	60.2 (56.3-64.1)	64.6 (63.4-65.8)
Insufficient activity	330	69.7 (66.2-73.2)	66.4 (65.5-67.4)	437	64.6 (61.1-68.0)	67.0 (65.9-68.1)
Sufficient activity	616	75.0 (72.1-77.8)	72.0 (71.1-72.8)	526	69.8 (67.5-72.2)	73.4 (72.3-74.4)
Not stated	42	63.8 (54.5-73.0)	65.1 (61.4-68.9)	30	69.9 (57.0-82.7)	67.9 (64.4-71.5)
**Vitamin D supplement**						
Took vitamin D	23	82.0 (72.3-91.6)	77.3 (74.2-80.3)	76	77.1 (71.4-82.8)	78.5 (76.7-80.3)
Took supplements but not vitamin D	290	72.0 (68.2-75.8)	67.4 (66.2-68.7)	366	63.0 (60.3-65.8)	66.7 (65.6-67.7)
Did not take supplements/not stated	851	72.5 (70.0-75.0)	69.4 (68.7-70.2)	808	66.3 (63.9-68.7)	69.5 (68.6-70.4)

*Not stated category not reported n ≤ 10. The weighting of data can lead to rounding discrepancies and totals not adding.

^†^Adjusted for age group, BMI categories, country of birth, season, income, education, smoking status, activity level, vitamin D supplement status.

The proportion of males, females and the population overall at various cutoff levels of serum 25(OH)D is shown in Table 3. Overall, 0.9% of the population had serum 25(OH)D below 25 nmol/L and 22.7% had levels below 50 nmol/L. When considered in the context of South Australian reported levels, 38.5% had serum levels below 60 nmol/L with 60.9% of participants in the optimal range of 60-160 nmol/L. There was a significantly higher proportion of females compared to males within each category (p < 0.05) except for those aged 75 years and over (p = 0.06).

**Table 3:** **Proportion of males, females and overall population below various cut off values for Vitamin D**

	Males		Females		Overall	
	n	% (95% CI)	n	% (95% CI)	n	% (95% CI)
Less than 25 nmol/L*	5	0.4 (0.2-1.0)	16	1.3 (0.8-2.2)	21	0.9 (0.6-1.4)
Less than 30 nmol/L*	18	1.6 (1.0-2.5)	43	3.5 (2.4-4.9)	61	2.5 (1.9-3.4)
Less than 40 nmol/L*	74	6.4 (4.9-8.3)	182	14.6 (12.1-17.5)	256	10.6 (9.1-12.4)
Less than 50 nmol/L*	215	18.5 (15.5-21.9)	334	26.8 (23.6-30.2)	549	22.7 (20.5-25.1)
Less than 60 nmol/L*	380	32.6 (28.9-36.6)	549	44.0 (40.2-47.8)	929	38.5 (35.8-41.3)
Less than 75 nmol/L*	692	59.5 (55.3-63.5)	861	68.9 (65.2-72.3)	1553	64.3 (61.6-67.0)
Less than 100 nmol/L	1005	86.4 (83.1-89.0)	1121	89.8 (87.4-91.7)	2127	88.1 (86.2-89.8)

The weighting of data can lead to rounding discrepancies and totals not adding.

*Chi square test indicates significant difference in prevalence in each category between males and females p < 0.05.

The prevalence of serum 25(OH)D levels below 50 and 75 nmol/L for males and females are shown in Tables 4 and 5 respectively. For males, those with vitamin D deficiency were more likely to be obese, not born in Australia and have a university level of education and those with levels < 75 nmol/L were more likely to be obese and had higher levels of education. Females with vitamin D deficiency were more likely to be obese and took supplements but not vitamin D or did not take supplements at all. Those with levels < 75 nmol/L were more likely to be obese and take supplements but not vitamin D. All of these participants also had blood samples taken in winter and spring. For both males and females, those who undertook a sufficient level of activity were less likely to have serum 25(OH)D less than 50 nmol/L or 75 nmol/L.

**Table 4:** **Prevalence of serum 25(OH)D levels < 50 and <75 nmol/l, and adjusted odds ratios (95% CI) in males**

	<50 nmol/l				<75 nmol/l	
***Variables***	***n***	***Prevalence***	***Odds ratio*** ^***†***^	***n***	***Prevalence***	***Odds ratio*** ^***†***^
**Overall**	215	18.5		692	59.5	
**Age (years)**						
24-34 years	54	21.4	1.00	147	58.5	1.00
35-44 years	41	16.0	0.73 (0.32-1.68)	157	61.8	1.20 (0.59-2.47)
45-54 years	45	18.8	0.75 (0.34-1.64)	155	64.5	1.27 (0.62-2.59)
55-64 years	33	17.6	0.58 (0.25-1.35)	110	58.3	0.89 (0.43-1.82)
65-74 years	16	12.8	0.37 (0.14-0.95)	64	52.6	0.80 (0.38-1.68)
75 years and over	26	24.4	0.85 (0.32-2.27)	59	54.8	0.90 (0.41-1.99)
BMI*						
Underweight/normal	40	15.3	1.00	141	54.2	1.00
Overweight	84	15.7	1.12 (0.60-2.12)	304	56.9	1.17 (0.74-1.83)
Obese	91	24.6	2.21 (1.19-4.11)	247	66.8	1.72 (1.05-2.80)
Country of birth*						
Australia	135	16.0	1.00	496	58.4	1.00
Other country	79	25.0	1.93 (1.22-3.05)	196	62.2	1.38 (0.95-2.01)
**Season**						
Summer	18	8.2	1.00	96	44.4	1.00
Autumn	32	11.7	1.75 (0.62-4.91)	136	49.0	1.37 (0.78-2.39)
Winter	90	26.0	4.55 (1.66-12.44)	225	65.2	2.76 (1.52-5.02)
Spring	75	23.1	4.08 (1.51-10.98)	235	72.3	3.90 (2.23-6.80)
**Income**						
Up to $60,000	96	20.8	1.00	261	56.4	1.00
More than $60,000	99	16.4	0.75 (0.43-1.30)	382	63.3	1.22 (0.81-1.84)
Don’t know/not stated	20	20.2	0.60 (0.23-1.57)	50	50.5	0.56 (0.23-1.37)
**Education**						
Some schooling	52	17.7	1.00	168	56.9	1.00
Completed high school	12	10.0	0.42 (0.16-1.08)	77	63.4	1.14 (0.61-2.14)
Apprentice/diploma	77	16.4	1.03 (0.63-1.70)	257	54.7	0.89 (0.60-1.33)
University or higher education	61	25.3	2.27 (1.17-4.37)	165	68.5	1.93 (1.03-3.62)
Refused/not stated	13	34.2	8.96 (1.21-66.45)	25	67.4	39.21 (3.09-496.76)
**Smoking status**						
Non/ex	167	17.9	1.00	554	59.1	1.00
Current	46	21.5	1.35 (0.76-2.39)	131	61.8	1.38 (0.82-2.33)
Not stated	2	13.1	0.93 (0.17-4.98)	7	46.9	0.92 (0.22-3.87)
**Physical activity**						
No activity	39	22.4	1.00	113	64.0	1.00
Insufficient activity	69	21.0	0.95 (0.55-1.63)	221	67.0	1.06 (0.66-1.71)
Sufficient activity	93	15.1	0.53 (0.31-0.90)	333	54.1	0.59 (0.38-0.91)
Not stated	13	30.6	0.20 (0.04-1.14)	25	60.4	0.05 (0.01-0.43)
**Vitamin D supplement**						
Took vitamin D	2	10.7	1.00	10	42.9	1.00
Took supplements but not vitamin D	43	14.8	2.13 (0.49-9.20)	182	62.7	2.88 (1.11-7.51)
Did not take supplements/not stated	170	19.9	2.93 (0.70-12.33)	500	58.8	2.17 (0.87-5.42)

*Not stated category not reported n ≤ 10. The weighting of data can lead to rounding discrepancies and totals not adding.

^†^Adjusted for age group, BMI categories, country of birth, season, income, education, smoking status, activity level, vitamin D supplement status.

**Table 5:** **Prevalence of serum 25(OH)D levels < 50 and <75 nmol/l, and adjusted odds ratios in females**

	<50 nmol/l				<75 nmol/l	
***Variables***	***n***	***Prevalence***	***Odds ratio*** ^***†***^	***n***	***Prevalence***	***Odds ratio*** ^***†***^
**Overall**	334	26.7		861	68.9	
**Age (years)**						
24-34 years	53	21.4	1.00	167	67.3	1.00
35-44 years	69	26.9	1.28 (0.60-2.70)	189	74.1	1.48 (0.74-2.95)
45-54 years	85	34.7	1.83 (0.92-3.66)	178	72.8	1.33 (0.70-2.56)
55-64 years	50	24.8	0.96 (0.45-2.04)	135	66.5	0.88 (0.45-1.76)
65-74 years	38	26.6	1.01 (0.45-2.27)	101	71.2	1.16 (0.55-2.45)
75 years and over	40	25.3	0.88 (0.37-2.10)	91	58.0	0.70 (0.32-1.54)
BMI*						
Underweight/normal	81	20.1	1.00	245	60.8	1.00
Overweight	99	24.4	1.29 (0.82-2.02)	272	67.0	1.41 (0.95-2.10)
Obese	154	35.0	1.97 (1.29-3.03)	344	78.1	2.32 (1.55-3.48)
Country of birth*						
Australia	233	25.3	1.00	624	67.7	1.00
Other country	100	31.0	1.26 (0.88-1.81)	234	72.4	1.35 (0.95-1.90)
**Season**						
Summer	48	16.1	1.00	186	63.0	1.00
Autumn	59	21.8	1.59 (0.86-2.94)	168	62.3	1.01 (0.62-1.63)
Winter	127	35.0	2.89 (1.67-5.01)	258	71.0	1.46 (0.91-2.36)
Spring	101	31.3	2.51 (1.46-4.29)	250	77.4	2.16 (1.32-3.55)
**Income**						
Up to $60,000	173	29.5	1.00	413	70.4	1.00
More than $60,000	129	24.8	0.90 (0.58-1.41)	364	69.9	0.90 (0.57-1.43)
Don’t know/not stated	33	22.8	0.81 (0.43-1.53)	84	59.1	0.72 (0.39-1.32)
**Education**						
Some schooling	144	28.7	1.00	341	67.9	1.00
Completed high school	44	24.0	0.82 (0.44-1.52)	129	70.3	1.03 (0.60-1.80)
Apprentice/diploma	85	27.7	1.04 (0.67-1.59)	217	71.0	1.07 (0.68-1.68)
University or higher education	52	22.7	0.92 (0.53-1.57)	156	68.1	1.20 (0.68-2.11)
Refused/not stated	9	32.2	2.59 (0.39-17.17)	17	61.4	4.11 (0.44-38.20)
**Smoking status**						
Non/ex	278	27.1	1.00	693	67.6	1.00
Current	52	25.1	0.86 (0.51-1.43)	159	75.9	1.43 (0.84-2.45)
Not stated	4	24.5	0.61 (0.23-1.65)	9	61.1	0.69 (0.25-1.90)
**Physical activity**						
No activity	95	37.0	1.00	193	75.4	1.00
Insufficient activity	135	30.9	0.75 (0.47-1.21)	314	71.9	0.81 (0.49-1.35)
Sufficient activity	96	18.3	0.37 (0.23-0.60)	336	63.9	0.59 (0.36-0.96)
Not stated	9	28.9	0.30 (0.05-1.79)	18	57.7	0.19 (0.02-1.60)
**Vitamin D supplement**						
Took vitamin D	6	8.3	1.00	46	60.4	1.00
Took supplements but not vitamin D	99	27.2	4.68 (2.03-10.75)	264	72.3	1.80 (1.02-3.17)
Did not take supplements/not stated	229	28.3	5.14 (2.31-11.45)	550	68.2	1.44 (0.86-2.44)

*Not stated category not reported n ≤ 10. The weighting of data can lead to rounding discrepancies and totals not adding.

^†^Adjusted for age group, BMI categories, country of birth, season, income, education, smoking status, activity level, vitamin D supplement status.

## Discussion

This study examined the levels of serum 25(OH)D within the population and although large studies have been undertaken in Australia and elsewhere (for example, Daly et al. [23], Melamed et al. [3], Green-Finestone et al. [10] and Brock et al. [29]), this study specifically examines a population sample from SA, weighted to be reflective of the state and with a wide range of covariates also measured, which allows an in depth examination of factors associated with vitamin D. Levels of vitamin D within the population have received increased focus over recent times due to the association of vitamin D deficiency with not only bone health but mortality and various other conditions [1–9]. As the majority of Australians obtain vitamin D as a result of exposure to sunlight, there is also the need to address vitamin D deficiency while ensuring that the risk of skin cancer and the skin cancer population messages are also recognised [1].

While the mean serum 25(OH)D levels within this population were within the optimal level recommended in SA, 38.5% still have levels below 60 nmol/L and 22.7% had levels below 50 nmol/L. The Australian Bureau of Statistics National Health Measures Survey (NHMS) conducted in 2011/12 also showed a similar proportion of South Australians (26.7%) aged 18 years and over with vitamin D levels < 50 nmol/L [30]. Additionally, there was a higher prevalence of deficiency among women (26.8%) compared to men (18.5%) which is similar to previous results [23]. However, debate does continue as to the optimal level of serum 25(OH)D, consequently some authors have recommended a level of ≥ 75 nmol/L is appropriate to ensure musculoskeletal health benefits [31]. In this study, approximately two thirds of participants (64.3%) had serum 25(OH)D below this level. However, these prevalences are below those obtained by Daly et al. [23] and may be due to differences in serum 25(OH)D assays, time of the year that samples were obtained and the population that was sampled. A prevalence of 22.7% with serum 25(OH)D levels below 50 nmol/L is consistent with results obtained from other Australian [18–22] and international studies [13–17], but also further highlights the impact of different study populations, locations, vitamin D intake and fortification of foods and methodology on study result on the importance of locally relevant data in order to determine the level of vitamin D deficiency.

Daly et al. [23] demonstrated that mean levels of 25(OH)D (unadjusted and adjusted) decreased with age for both males and females and that the prevalence of deficiency generally increased with age. However in this study, age was not significantly associated with vitamin D deficiency and mean levels of 25(OH)D, were higher in the older age groups. This study also examined the use of vitamin D supplements. The non-use of vitamin D supplements was significantly associated with 25(OH)D deficiency among females but not males. While only a small proportion (4.1% overall) reported using supplements, use did increase across age groups with the highest proportion of those taking vitamin D, in the 75 years and over age group, where mean levels of 25(OH)D were also higher, perhaps demonstrating the effectiveness of supplement use amongst the older population in South Australia. These results compare well with the NHMS which demonstrated that 4.3% of the population of SA aged 18 years and over, took vitamin D supplements in 2011/12 [32] and that use was more common among older women [33]. However, compliance and tolerance can be issues within the older population [2] and the elderly, particularly those in residential care have been identified as a high-risk group for vitamin D deficiency [34]. These findings highlight that taking vitamin D may be effective in achieving higher mean levels and reducing vitamin D deficiency but improved recommendations relating to supplementation may be required in order to increase the rate of uptake.

Also of note is the issue of assay technique, which can exhibit variations. There has also been a recent increase in the number of tests for 25(OH)D, thus traditional test procedures have been discarded for tests that are more prone to interference [35]. Both of the foci described above align well with quality use of medicines and diagnostic testing messages.

The effect of seasonality on 25(OH)D levels is highlighted in this study. Both adjusted and unadjusted mean values of serum 25(OH)D were lower in winter and spring as were the adjusted and unadjusted prevalences of deficiency. Participants with samples taken in both winter and spring were more likely to have vitamin D levels less than 50 and 75 nnmol/L. These findings support previous results [1, 10, 23, 25] and highlight that season is a major factor in determining vitamin D levels. Latitude has also been shown to be consideration associated with lower levels of serum 25(OH)D in Australia [22, 23] and while the combination of these two factors only accounted for approximately one fifth of the variation in vitamin D levels [22] it may be that a more active vitamin D supplementation program aimed at increasing the number of people taking supplements needs to occur, in winter and spring.

Mean vitamin D levels were generally higher among those with lower education levels, which may reflect occupational choices and similarly to Daly et al. [23], males with higher education levels were more likely to have 25(OH)D levels less than 50 nmol/L, whereas females were not. This may reflect age, supplement use and occupational influences among both males and females. Country of birth also influenced vitamin D levels with males not born in Australia more likely to be vitamin D deficient. Ethnicity has previously also been shown to be a factor associated with lower levels of vitamin D [10, 23] and has been linked to darker skin, time spent outdoors and clothing particularly among females [1, 10, 23]. In this study, while males not born in Australia were more likely to have vitamin D levels < 50 nmol/L, the effect was of country of birth was not as marked for females.

This study identified that low levels of physical activity were associated with low levels of serum 25(OH)D; those with higher levels of physical activity were less likely to have vitamin D levels below 50 or 75 nmol/L. This is in line with the findings of Daly et al. [23] and Brock et al. [29], who demonstrated that vigorous activity was a predictor of vitamin D status. Generally it is considered that high levels of activity are a surrogate measure of sun exposure, however it remains unclear whether exercise itself may contribute to vitamin D status [29]. High levels of BMI were also associated with low vitamin D levels with both males and females classified as obese more likely to be vitamin D deficient. Those who are obese may tend to undertake less activity, however this inverse relationship has also been attributed to cholecalciferol, the precursor to vitamin D being trapped in adipose tissue and thus the contribution to whole body vitamin D status is minimized as conversion to 25(OH)D does not occur [29].

This study demonstrated that non and ex-smokers had higher mean levels of serum 25(OH)D compared to current smokers for both males and females, as has been shown in previous studies [23, 36] however current male smokers were not significantly more likely to be vitamin D deficient and current female smokers had a lower prevalence of serum 25(OH)D less than 50 nmol/L compared to non-smokers possibly as a result of requirements to smoke outdoors in some environments. Gender, obesity, season, activity levels and vitamin D supplementation all appear to have a stronger association with vitamin D deficiency in this cohort than smoking status.

The strengths of this study are the large sample size of over 2000 blood samples which were available to be tested for serum 25(OH)D and the range of behavioral measurements obtained as part of the NWAHS. Weighting of the data ensures that the results are representative of the population from which the original sample was randomly selected. Limitations include the self-reporting of health risk factors such as smoking and physical activity, the small number of participants born in countries other than Australia and the use of country of birth as an indicator of ethnicity and the non-measurement of factors such as parathyroid hormone and bone resportion markers which would enable a greater understanding of the role of 25(OH)D in relation to bone and mineral homeostasis in this cohort. Information relating to the use of sunscreen and the time spent outdoors was also not collected, however both of these factors may also impact vitamin D levels. Australia has, however, a high rate of melanoma compared with other countries and there has been a focus on skin cancer prevention activities with a resultant increase in sun protection behaviours which may impact on the prevalence of vitamin D deficiency [37]. The use of foods fortified with vitamin D was also not specifically examined.

## Conclusions

In conclusion, vitamin D levels below 50 nmol/L are prevalent in the South Australian population, and given the burden of conditions related to low levels of vitamin D, represent a significant public health issue. Targeting of physical activity and reduction in obesity may assist in improving vitamin D levels. The use of supplementation remains an issue at a population level and in line with a quality use of medicines approach [38] consideration should be given to alternative methods of supplementation provision in order to improve serum 25(OH)D levels.
